# Changes in Lipids and Proteins of Common Carp (*Cyprinus carpio*) Fillets under Frozen Storage and Establishment of a Radial Basis Function Neural Network (RBFNN)

**DOI:** 10.3390/foods12142741

**Published:** 2023-07-19

**Authors:** Chunli Kong, Caiping Duan, Yixuan Zhang, Ce Shi, Yongkang Luo

**Affiliations:** 1 School of Food and Health, Beijing Engineering and Technology Research Center of Food Additives, Beijing Advanced Innovation Center for Food Nutrition and Human Health, Beijing Technology and Business University, Beijing 100048, China; kongc0606@btbu.edu.cn (C.K.);; 2 Beijing Laboratory for Food Quality and Safety, College of Food Science and Nutritional Engineering, China Agricultural University, Beijing 100083, China; 3 Information Technology Research Center, Beijing Academy of Agriculture and Forestry Sciences, Key Laboratory of Cold Chain Logistics Technology for Agro-Product, Ministry of Agriculture and Rural Affairs, Beijing 100097, China

**Keywords:** common carp, frozen storage, lipid oxidation and hydrolysis, endogenous enzyme activity, protein denaturation, RBFNN

## Abstract

Storage via freezing remains the most effective approach for fish preservation. However, lipid oxidation and protein denaturation still occur during storage, along with nutritional loss. The extent of lipid alteration and protein denaturation are associated with human health defects. To precisely predict common carp (*Cyprinus carpio*) nutritional quality change during frozen storage, here, we first determined lipid oxidation and hydrolysis and protein denaturation of common carp fillets during 17 weeks of frozen preservation at 261 K, 253 K, and 245 K. Results showed that the content of thiobarbituric acid reactive substances (TBARS) and free fatty acids (FFA) were significantly increased. However, salt-soluble protein (SSP) content, Ca^2+^-ATPase activity, and total sulfhydryl (SH) content kept decreasing during frozen storage, with SSP content decreasing by 64.82%, 38.14%, and 11.24%, respectively, Ca^2+^-ATP enzyme activity decreasing to 12.50%, 18.52%, and 28.57% Piμmol/mg/min, and SH values decreasing by 70.71%, 64.92%, and 56.51% at 261 K, 253 K, and 245 K, respectively. The values at 261 K decreased more than that at 253 K and 245 K (*p* < 0.05). Ca^2+^-ATPase activity was positively correlated (r = 0.96) with SH content. Afterwards, based on the results of the above chemical experiments, we developed a radial basis function neural network (RBFNN) to predict the modification of lipid and protein of common carp fillets during frozen storage. Results showed that all the relative errors of experimental and predicted values were within ±10%. In summary, the quality of common carp can be well protected at 245 K, and the established RBFNN could effectively predict the quality of the common carp under frozen conditions at 261–245 K.

## 1. Introduction

Common carp (*Cyprinus carpio*), one oldest freshwater fish species, is artificially cultured for commercial use around the world, due to its high breeding efficiency, short growth period, and easy cultivation [1,2]. The global production of carp in 2018 was 28.9 million tons, which accounts for 25.2% of the global aquaculture production [3]. Although fresh fish is usually the first choice for consumers, due to geographical reasons, there are still about 62% of fishes consumed after the freezing process [4]. The recent COVID-19 pandemic has also seen an increasing intake of frozen or preserved fish, while a decreasing intake in fresh fish [5]. Thus, studies on frozen fish deserve more attention.

Freshwater fish are abundant in nutrients that are digestible, absorbable, but also highly perishable. Freezing preservation is still the most effective approach commonly used to inhibit fish deterioration caused by spoilage microorganisms and indigenous enzymes [6]. However, lipid oxidation [7], hydrolysis [8], and protein denaturation [9] occur during the frozen storage. Endogenous and microbiological hydrolytic enzymes such as proteases, lipases, phospholipases, peroxidases, and lipoxygenases are still detrimental factors causing fish meat deterioration during frozen storage [10,11,12]. Hematyar et al. [13] reported that there was a gradual increase in thiobarbituric acid reactive substances (TBARS) content in *Cyprinus carpio* muscle within 6 months of frozen storage, which indicated that there was intensive oxidation of lipid. Once oxidation began, the reaction accelerated oxidation of sensitive substances [14]. Even under frozen storage, the lipid oxidation can also be accelerated by the formation of free fatty acids (FFA) [15], causing deterioration of Ca^2+^-ATPase, denaturation of protein [6], and aggregation of myofibrillar proteins [16].

With frozen storage progressing, ice crystallization formation exerts adverse effects on fish matrix structure, lowers sensory acceptance, and nutritional loss [17,18]. The alteration of lipid and denaturation of protein during frozen storage are other main factors reducing fish’s nutritional value. As suggested, a TBARS content below 0.58 mg/kg was considered as fresh for human consumption, while values between 0.58 and 1.51 mg/kg were considered as moderate corruption [19]. The consumption of fish that contain oxidised lipids might be associated with chronic disease risks. For example, increased TBARS levels in serum might be a biomarker trait for depression [20]. FFA, at a relatively low level of 0.05 mmol/L, can already induce hepatic steatosis in human HepG2 cell lines in vitro [21]. Furthermore, FFA has been considered as “danger signals”, which can be captured by Toll-like receptor 2/4, priming metabolic stress of adipose inflammation and insulin resistance induced by obesity and mediated by NOD-like receptor protein 3 (NLRP3) inflammasome [22,23]. Therefore, suitable predictive technology must be developed to better understand fish safety, quality, and nutritional value before they are purchased by consumers [24].

Traditionally, the nutritional quality of fish is estimated by physical and chemical experiments, which are time-consuming and harmful to both of the environment and the human body. In last decades, mathematical models have been widely established for quality prediction of foods [25,26]. When the model is developed, quality of food can be calculated at certain storage times and temperatures based on the initial values without conducting complex experiments. Chen et al. [27] developed Arrhenius and Random Forest models to predict *Litopenaeus vannamei* fillets quality under chilled storage, and Random Forest model showed better reliable goodness of fit. In the prediction of quality for *Solenocera melantho* during frozen storage [28], the Arrhenius model did not predict quality accurately. Wang et al. [26] found that compared to the Arrhenius model, the artificial neural network (ANN) exhibited higher precision for quality prediction of *Oreochromis niloticus* at different temperatures. RBFNN is one type of feed-forward neural network with optimum approximation performance and global optimal features. It has been widely applied in data classification [29,30] and predictive control [31,32]. However, there are few reports on the development of RBFNN for quality prediction of freshwater fish during frozen storage.

Better understanding of the fish nutrition during frozen storage may provide suggestions to consumers and aquatic industry. Here, we first performed chemical experiments to determine changes in lipid and protein of common carp fillets under different frozen temperatures by measuring TBARS, FFA, SSP, Ca^2+^-ATPase activity, SH content, then correlations between changes in lipid and protein were explored, and finally a predictive model was developed based on the quality indicators listed above for common carp under frozen preservation method with RBFNN.

## 2. Materials and Methods

### 2.1. Materials

Sixty living common carp [mean weight (1398.36 ± 116.32 g), mean length (43.87 ± 0.71 cm)] were obtained from an aquatic products wholesale market (Beijing, China) and were transported to the laboratory alive, in aerated boxes containing water. The fish were sacrificed by stunning, and fillets were taken after washing with flowing water. Then, the fish fillets were packed in valve bags after draining for 10 min, and were distributed equally among 261 K, 253 K, and 245 K, respectively. Three fillets were taken randomly from each temperature 5 h later (after they were completely frozen) for the determination of initial values of all the indicators. For the 17 weeks’ experiment (based on sensory evaluation), analysis was taken weekly in the first three weeks, and then it was carried out twice a week. Before each analysis, three carp fillets (*n* = 3) were chosen at random from each of the three temperature groups and thawed at 4 °C for 24 h. After thawing, each fillet was minced individually using a meat Grinder (BH824, Jinzheng Kitchen & Sanitary Equipment Co., Ltd., Zhongshan, China) for later determination.

### 2.2. Methods

#### 2.2.1. Thiobarbituric Acid Reactive Substances (TBARS)

The content of TBARS was conducted with the method of Noori [33] with a slight modification. Two grams of minced samples were mixed with trichloroacetic acid (TCA) (16 mL, 5%, *w*/*v*) and BHT-ethanol (100 μL, 2 g/L) at equal speeds for 2 min. The homogenization was then centrifuged, and the supernatant (5 mL) was mixed with TBA (1 mL, 0.01 M). After boiling in water for 40 min, absorbance was measured at 532 nm. Distilled water was taken as blank. The value of TBARS was calculated by multiplying 10.2 (It is obtained from the standard curve of 1,1,3,3-tetraethoxypropane, the precursor of malondialdehyde, which is equivalent to the mass of malondialdehyde analogues per kilogram of fish) and calculated as malonaldehyde/sample (mg/kg).

#### 2.2.2. Free Fatty Acids (FFA)

Crude fat was extracted by a modified Kong method [34]. The minced carp fillets, weighing around 30 g, were rapidly homogenized for 2 min using a mixture of 30 mL chloroform and 60 mL methanol. This was followed by the addition of 30 mL of chloroform and 30 mL of distilled water, which were sequentially incorporated and homogenized for 30 s. The extraction was then collected by suction filtration, transferred into a separating funnel, and left to stand for 3 h until the mixture had been completely separated. The organic phase in the bottom was received and dried under nitrogen flow. Before analyzing, the obtained crude fat was placed in a desiccator for 24 h.

FFA content was determined based on Shi and Lei [35]. The crude fat sample (0.05–0.10 g) was dissolved in a solution containing 5 mL of toluene and 1 mL of cupric acetate-pyridine reagent (5%, *w*/*v*, pH 6.0). After oscillating the mixture for 2 min, it was centrifuged at 3000 rpm for 5 min. The absorbance of the resulting supernatant was measured at 715 nm using a spectrophotometer (Unico Instrument Co., Ltd., Shanghai, China). The mass of FFA was determined through the standard curve of FFA made with oleic acid, and the content of FFA was expressed as g FFA/100 g of lipids.

#### 2.2.3. Salt-Soluble Protein (SSP)

SSP was extracted according to Liu [36], with some modifications. Accurately weighed 2 g of chopped carp fillet samples were homogenized in an ice bath and the precipitate was collected by centrifugation at 10,000 rpm for 10 min at 277 K. The washed precipitate was homogenized with 30 mL Tris maleate buffer (0.6 M NaCl-20 mM Tris maleate, pH 7.0) for 10 s, followed by and extracted at 277 K for 2 h.

The content of SSP was measured according to Yu et al. [37]. In short, SSP extract (1 mL) was added in Biuret protein assay (4 mL) and reacted for 20 min at room temperature. The absorbance was read at 540 nm using a spectrophotometer. The content of SSP was calculated as protein/minced carp fillets (mg/g).

#### 2.2.4. Ca^2+^-ATPase Activity

The myofibril protein solution was prepared as follows: The supernatant from an 8 mL sample of the SSP extract was removed by being washed twice with chilled deionized water. The resulting sediment was then dissolved in NaCl (3 mL, 0.6 M). The myofibril protein solution’s concentration was assessed following the methodology outlined by Yu et al. [37] and was diluted to achieve a concentration of 4 mg/mL.

Ca^2+^-ATPase activity was measured following the approach described by Lin et al. [38]. The reaction system was initiated by adding ATP (0.2 mL, 20 mM) to a combination of myofibril protein solution (0.4 mL), Tris-maleate buffer (0.2 mL, 0.5 M, pH 7.0), CaCl_2_ solution (0.2 mL, 0.1 M), and deionized water (3 mL), which was conducted at 298 K. Three minutes later, the reaction was ended by the introduction of TCA (2 mL, 15%, *w*/*v*). Then, the reaction system was subjected to centrifugation at a speed of 10,000 rpm for a duration of 2 min, following which the absorbance of the resulting supernatant was measured at a wavelength of 640 nm. For the blank, prior to the addition of ATP, the reaction was ended by the addition of TCA. The activity of Ca^2+^-ATPase was quantified as phosphate/mg protein (μmol/min).

#### 2.2.5. Total Sulfhydryl (SH) Content

The content of total SH was determined in accordance with the experimental procedure established by Zheng et al. [39], with a minor modification. A 0.5 mL sample of 4 mg/mL myogenic fibronectin solution was mixed with 4.5 mL of buffer A (8 M urea, 2% SDS, 10 mM EDTA, pH 8.0), and then 0.5 mL of buffer B (0.1% DTNB-Tris-HCl) was added to the 4 mL reaction mixture and incubated in water at 313 K for 25 min. The spectrophotometer was used to measure the absorbance at a wavelength of 412 nm. For the blank, the protein sample was replaced by 0.6 M of NaCl. The SH content was calculated using the following equation:

SH content (mol/10^−5^ g) = A × D/B × C (1)

where A represents the absorbance, B represents the concentration of myofibrillar protein (4 mg/mL), C denotes the molar extinction coefficient (13,600 M^−1^cm^−1^), and D signifies the dilution factor, 11.25.

#### 2.2.6. Radial Basis Function Neural Networks (RBFNNs)

**Figure 1:**
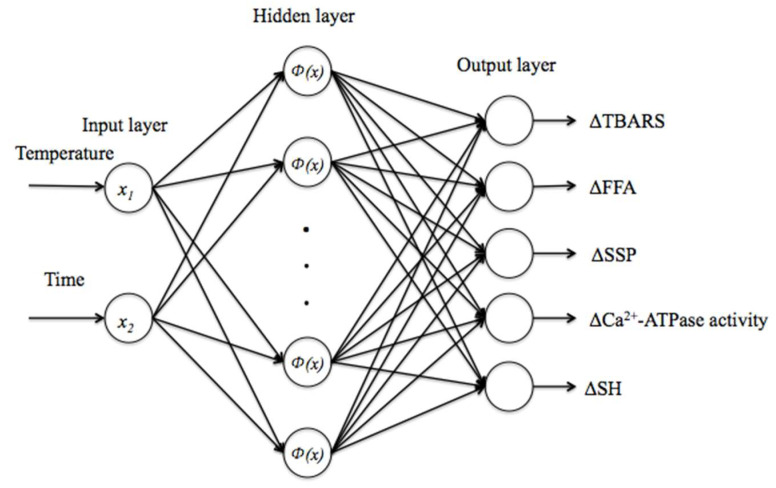
Structure of RBFNN establishment.

In this study, the RBFNN architecture utilized a three-layer network with a single hidden layer (Figure 1) and was established through Matlab R2013b. Neurons of the input layer are responsible for the collection of incoming information and transfer them to the high dimensional hidden space. The input layer of the RBFNN comprised two neurons representing storage temperature (K) and time (week). The hidden layer is an internal information processing layer. It provides a collection of nonlinear functions [Φ(x)] that are in charge of information transformation and processing. Finally, the information was passed on to the output layer where the linear relationship output was completed, and this is called the self-organized learning stage. Connections between neurons in distinct layers were established through the utilization of weights and biases. During training of the network, if the actual output was not inconsistent with the expected output, the process entered erroneous reversed dissemination stage. Weights among the three layers would be adjusted on and on until they satisfied the error requirement. Here, the output layer consisted of five neurons, representing ΔTBARS, ΔFFA, ΔSSP, ΔCa^2+^-ATPase activity, and ΔSH. The calculation for ΔC was performed in the following manner:

ΔC = C_t_ − C_0_ (2)

in which C_t_ represents the experimental values of TBARS, FFA, SSP, Ca^2+^-ATPase activity and SH at time t, and C_0_ is the initial values of each indicator.

The output vector of the neurons located in the *j*th hidden layer was calculated as:

(3) $$Y_{j}=exp\left[-{\left[\sqrt{\sum_{j=1}^{n}{\left[w_{\mathrm{ji}}-x_{i}\right]}^{2}}\times w_{j0}\right]}^{2}\right]=\exp\left[-0.8326^{2}\times {\left[\frac{∥w_{\mathrm{ji}}-x_{i}∥}{C_{i}}\right]}^{2}\right]$$

The expression for the output vector of neurons in the *k*th output layer can be written as:

(4) $$Y_{k}=\sum_{k=1}^{N}w_{kj}Y_{j}+w_{k0}=\sum_{k=1}^{N}w_{kj}\exp\left[-0.8326^{2}\times {\left[\frac{∥w_{\mathrm{ji}}-x_{i}∥}{C_{i}}\right]}^{2}\right]+w_{k0}$$

where *Y_j_* represents the output vector of neurons in *j*th hidden layer, *n* denotes the total number of hidden neurons, *w_ji_* signifies the weights associated with input to hidden neurons, *w_j0_* denotes the bias of input to hidden neurons, *x_i_* represents the *i*th input vector, *C_i_* corresponds to 0.8326/spread, *Y_k_* signifies the *k*th output vector of the output layer, *N* represents the total number of output neurons, *w_kj_* signifies the weights associated with output to hidden neurons, *w_k0_* denotes the bias of output to hidden neurons.

During the training process, the variables in the input layer were normalized to −1 and 1, similarly to the output layer. The overall predictive effect of the network was estimated using mean square error (MSE) and regression coefficients (R^2^) between the experimental and predictive values. In this study, the network was trained with 0, 2, 4, 6, 8, 10, 12, 14, 16, 18, 20, 22, 24, 26, and 28 neurons in the hidden layer, and 0.05, 0.50, 1.00, 1.50, 2.00, and 2.50 of spread to obtain the minimum MSE and to ascertain the optimal number of neurons in the hidden layer [40].

Experimental values of TBARS, FFA, SSP, Ca^2+^-ATPase activity, and SH at 261 K, 253 K, and 245 K were used to develop RBFNN. To assess the predictive performance of RBFNN, the data of 253 K were utilized, and the relative errors between the experimental (C_exp_) and predictive (C_pre_) values at 253 K were employed as validation metrics:

Relative error (%) = abs ((C_exp_ − C_pre_)/C_exp_) × 100 (5)

### 2.3. Statistical Analysis

All measurements were conducted in triplicate. Experimental data were analyzed by Microsoft^®^ Excel^®^ version 2011 and Matlab R2013b. Analysis of variance (ANOVA) was performed using Compare Procedure of SPSS 20.0. The average values were compared using the least significant difference (LSD) procedure, with a significance level set at *p* < 0.05.

## 3. Results and Discussion

### 3.1. Thiobarbituric Acid Reactive Substances (TBARS)

**Figure 2:**
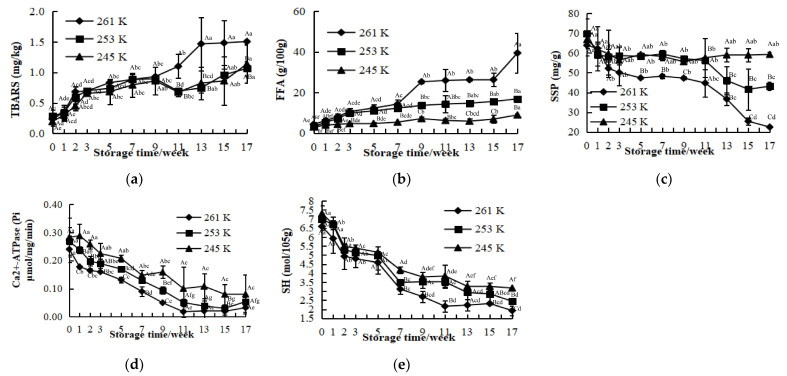
Changes in TBARS (**a**), FFA (**b**), SSP (**c**), Ca^2+^-ATPase activity (**d**), and SH content (**e**) of common carp stored at 261 K, 253 K, and 245 K. a–g. Different letters of the same storage temperature stands for significant difference (*p* < 0.05); A–C. Different letters of the same storage time stands for significant difference (*p* < 0.05).

TBARS were the secondary breakdown products of lipid oxidation that was initiated by enzymatic and non-enzymatic reactions [41]. The rate and extent of oxidation were related to the species of fish [42], storage conditions [43], and saturation degree of fatty acids [44]. Ice crystals formed during frozen storage would pierce cells and released pro-oxidants [45,46], potentially promoting oxidation. In this study, content of TBARS exhibited an increase during the frozen storage, which can be seen from Figure 2a. During the first 9 weeks, no significant difference was observed (*p* > 0.05) among the three temperatures, which reached 0.93 ± 0.02, 0.89 ± 0.04, and 0.86 ± 0.23 mg/kg at 261 K, 253 K, and 245 K, respectively. Thereafter, content of TBARS at 261 K increased consistently up to the end of the storage, while it showed a slight decrease and then increased gradually for the samples at 253 K and 245 K. The observation was in agreement with the study of the changes of content of TBARS (increased from 0.1 to 0.6 mg MDA/kg meat) during frozen preservation of fish reported by Li et al. [47]. At the 17th week, content of TBARS rose to 1.06 ± 0.11 and 1.14 ± 0.32 mg/kg at 253 K and 245 K, respectively, indicating it was moderately rancid [19], whereas a similar concentration at 261 K was already reached during the 11th week. The increase in content of TBARS indicated intensive oxidation of lipid during frozen storage, and in comparison to the samples stored at 261 K, the relatively lower concentration of TBARS at 253 K and 245 K could account for the effective inhibition of lipid oxidation.

### 3.2. Free Fatty Acids (FFA)

FFA are commonly employed as indicators to assess the extent of lipid hydrolysis. Fish lipids are more prone to oxidation and hydrolysis due to their high content of polyunsaturated fatty acids (PUFA) and strong autolytic activity [15]. FFA values under different frozen temperatures with storage are presented in Figure 2b. No significance was observed for the initial FFA levels (4.33 ± 0.93, 3.08 ± 0.09, and 2.99 ± 0.59 g/100 g at 261 K, 253 K, and 245 K, respectively), while it increased to various degrees for the three treatments. FFA concentration increased gradually at 253 K and 245 K during the whole storage, whereas it showed a jump for 261 K at the 7th and the 15th week. A different tendency was obtained by Tanaka et al. [48], who reported that FFA concentration started to increase after the 3rd month at 253 K for the 12 month storage of *Scomber japonicus*. However, there was already a significant increase in FFA content for *Mytilus galloprovincialis* stored at 253 K for 10 days [49]. These may indicate a species dependent effect. In addition to species, preservation methods such as brine showed a lower response of FFA content during frozen storage according to what was reported previously [34]. In the end, the content of FFA at 261 K was elevated to 39.46 ± 9.89 g/100 g, which was 2.35 and 4.42 times as high as the concentration at 253 K and 245 K. Also, FFA concentration at 261 K was consistently more than that at 253 K and 245 K. These results indicated that FFA still formed during freezing, but the hydrolysis of lipids was significantly suppressed at lower temperatures [8].

### 3.3. Salt-Soluble Protein (SSP)

The content of SSP is an important index for the evaluation of myofibrillar protein denaturation of fish under process or during storage [50]. Figure 2c illustrates alterations in SSP for all samples during the frozen storage. The initial concentrations of SSP were 63.84 ± 5.12, 69.74 ± 7.57, and 66.74 ± 2.72 mg/g at 261 K, 253 K, and 245 K, respectively. As storage time progressed, SSP concentration decreased gradually, and the values of SSP at 261 K were significantly lower than that of 253 K and 245 K at the same stage (*p* < 0.05). At the 13th week, there was a dramatic decrease in SSP at 261 K and 253 K (*p* < 0.05), while the concentration of SSP at 245 K kept fluctuating throughout the entire duration of the frozen conditions (*p* > 0.05). At the 17th week, the contents of SSP were 22.46 ± 0.47, 43.14 ± 2.07, and 59.24 ± 1.26 mg/g at 261 K, 253 K, and 245 K, with a decrease of 64.82%, 38.14%, and 11.24%, respectively. A similar result was obtained by Shi and Yang [51], where the protein extractability of *Micropterus salmoides* at 255 K decreased to a greater extent than that at 233 K and 193 K during 1 month of storage. The decrease in SSP might be due to the aggregation caused by secondary interactions and disulfide bridges [46,51]. Even under frozen storage, the structure of fish muscle can be disrupted, causing enzyme release from lysosomal vesicles, subsequently with protein aggregation and proteolysis, and by which the protein extractability lowered as the freezing temperature increased [51,52]. Also, the aggregation of protein is temperature dependent [53]. In this research, the relatively slower rates of decrease in SSP at 253 K and 245 K were probably because lower frozen temperatures protected the structures of the protein effectively and inhibited the aggregation.

### 3.4. Ca^2+^-ATPase Activity

The initial values of Ca^2+^-ATPase activity were 0.24 ± 0.05, 0.27 ± 0.05, and 0.28 ± 0.09 Pi μmol/mg/min at 261 K, 253 K, and 245 K, respectively, as shown in Figure 2d. With frozen storage progressed, a substantial decrease (*p* < 0.05) of Ca^2+^-ATPase activity was observed during the first 11 weeks, but it tended to slow down thereafter. The values at 245 K were consistently higher than those at 261 K and 253 K. Compared to the initial values, Ca^2+^-ATPase activity decreased to 12.50%, 18.52%, and 28.57% Pi μmol/mg/min at 261 K, 253 K, and 245 K at the end of storage, respectively. Sriket et al. [54] showed a similar tendency of Ca^2+^-ATPase activity to decrease in basa (*Pangasius bocourti*) fillets during frozen storage. However, this was different from the results reported by Wu et al. where the activity of Ca^2+^-ATPase was completely lost at 8 weeks of storage of *Aristichthys nobilis* fish fillets at 263 K and 253 K, and at 10 weeks storage at 243 K, indicating fish species dependence [55]. Ca^2+^-ATPase, which is a type of transmembrane enzyme, is located at the globular head of myosin, and it makes up half of the myofibrillar protein, which represents the major protein component in fish muscle [56]. Thus, the inactivation of Ca^2+^-ATPase activity can be attributed to the denaturation of proteins. Moreover, the sulfhydryl group present in cysteine serves as the active center of Ca^2+^-ATPase, indicating that the decline in Ca^2+^-ATPase activity could also be linked to the oxidation of sulfhydryl group [57]. Findings from the study demonstrated that high Ca^2+^-ATPase activity at 245 K suggested that the integrity of the globular head was well protected, as well as the lower denaturation of protein.

### 3.5. Total Sulfhydryl (SH) Content

The sulfhydryl group tends to be oxidized to the disulfide bond because of its strong reactivity, which can destroy the protein structure and modify its physicochemical and functional properties further [58,59]. Changes in SH content under different frozen temperatures can be seen in Figure 2e. During the first 7 weeks, SH content decreased dramatically (*p* < 0.05) from 6.59 ± 0.20, 6.98 ± 0.42, and 7.31 ± 0.43 mol/10^5^ g to 3.12 ± 0.28, 3.49 ± 0.16, and 4.18 ± 0.19 mol/10^5^ g at 261 K, 253 K, and 245 K, respectively. After 7 weeks, SH content at 261 K kept decreased noticeably (*p* < 0.05) until the 11th week, while the values at 253 K and 245 K showed a gradual decrease until the end of storage (*p* < 0.05). Gao et al. [60] also reported a similar tendency of SH content to decrease (rapidly in the first 4 weeks, followed by a less extent afterwards) during frozen storage of bighead carp. Differently, oxygen isolation, i.e., vacuum packaging, of bighead carp fillets caused a constant gradual decrease in SH content during frozen storage [61]. The decrease in SH content might be attributed to the disruption of protein integrity. As we observed, the degradation of myofibrillar proteins still occur under frozen temperature. Subsequently, the N-terminal structures of proteins was uncovered and more prone to oxidation [61]. The content of SH was decreased and could be attributed to the formation of disulfide bonds as a result of the oxidation of sulfhydryl groups or interactions of disulfide bonds [46]. Towards the end, the contents of SH decreased by 70.71%, 64.92%, and 56.51% at 261 K, 253 K, and 245 K, respectively, which indicated that the oxidation of SH groups was significantly inhibited under lower freezing temperatures.

### 3.6. Relationship between TBARS, FFA, SSP, Ca^2+^-ATPase, and SH Content

A strong positive correlation was observed between content of TBARS and FFA (r = 0.82), and among SSP, Ca^2+^-ATPase, and SH content (Table 1). The content of TBARS and FFA were negatively correlated with SSP, Ca^2+^-ATPase, and SH. As stated above, the content of TBARS and FFA both significantly increased with frozen storage time, indicating that the hydrolysis and oxidation of lipids may mutually promote the values as reported in other studies [8,47]. Differently, the content of SSP, Ca^2+^-ATPase, and SH were all significantly decreased under frozen storage as shown above. The Pearson’s positive correlations of SSP, Ca^2+^-ATPase, and SH further demonstrated the disruption of common carp protein integrity and the positive association between protein solubility and structure integrity [62,63].

### 3.7. Establishment and Validation of RBFNN

The RBFNN model was developed using experimental data of TBARS, FFA, SSP, Ca^2+^-ATPase, and SH at 261 K, 253 K, and 245 K. A random sample of 70% of the data was used for model development, 15% of the data for assessment, and the remaining data to determine the fitting results. According to the data presented in Table 2, MSE decreased with the increase in neurons in the hidden layer, reaching the minimum value (0.00006) with a spread of 0.50. Considering the proper fitting extent, 28 neurons in the hidden layer were accepted. Thus, a neural network model based on RBFNN was constructed, utilizing 28 neurons in the hidden layer. The detailed information on the neuron weights and algorithms used can be found in Supplementary Material.

In order to validate the predictive accuracy of the RBFNN, relative errors between experimental and predictive values at 253 K were measured (Table 3). As mentioned by Kaymak-Ertekin and Gedik [64], models were considered acceptable if the relative errors fell within the range of ±10%. The relative errors between experimental and predictive values of TBARS, FFA, SSP, Ca^2+^-ATPase, and SH content at 253 K were all within ±10%. Apart from the value (−9.18%) of Ca^2+^-ATPase at the 17th week, all the others were within ±6%, which indicated the high predictive accuracy of RBFNN. To evaluate the overall fitting performance of every indicator at various temperatures, the MSE and R^2^ were computed by comparing the experimental and predicted values (Table 4). Extremely low MSE and high R^2^ demonstrated the excellent overall fitting performance of the established RBFNN.

## 4. Conclusions

In this study, quality changes in lipid oxidation and hydrolysis and protein denaturation of common carp fillets were studied under different frozen storage. Results showed that the deterioration of lipids had a negative correlation with the denaturation of protein based on the high Pearson’s correlations. The quality of common carp can be well protected at 245 K, as indicated by the relatively low concentration of TBARS and FFA and the high values of SSP, Ca^2+^-ATPase, and SH. Furthermore, RBFNN was established based on these indicators with acceptable relative errors, which proved that RBFNN could be a very promising method for the prediction of quality changes in common carp fillets under frozen conditions of 261–245 K. However, the indicators here may not fully reflect the quality changes of common carp fillets during frozen storage, and other quality parameters can also be considered, such as spoilage microorganisms, pH values, total volatile basic nitrogen, and bioamines. As lipid hydrolysis and oxidation are highly influenced by the saturation degree of fatty acids, the fatty acids profile of common carps is also suggested to be included for further investigation.

## Figures and Tables

**Table 1:** Pearson’s correlation (r) between TBARS, FFA, SSP, Ca^2+^-ATPase, and SH content of common carp during frozen storage.

Indicators		TBARS	FFA	SSP	Ca^2+^-ATPase	SH
TBARS	Person’s correlation	1	0.82 **	−0.84 **	−0.85 **	−0.91 **
FFA	Person’s correlation		1	−0.90 **	−0.77 **	−0.76 **
SSP	Person’s correlation			1	0.74 **	0.74 **
Ca^2+^-ATPase	Person’s correlation				1	0.96 **
SH	Person’s correlation					1

** Significance level at 0.01 (2-tailed).

**Table 2:** Values of MSE for TBARS, FFA, SSP, Ca^2+^-ATPase activity, and SH content prediction under different spreads and neurons in the hidden layer.

Neurons	MSE	Neurons	MSE	Spread	MSE
0	0.26884	16	0.00638	0.05	0.00191
2	0.13414	18	0.00488	0.10	0.00014
4	0.07946	20	0.00313	0.50	0.00006
6	0.04552	22	0.00204	1.00	0.00011
8	0.02378	24	0.00147	1.50	0.00020
10	0.01771	26	0.00077	2.00	0.00059
12	0.01144	28	0.00006	2.50	0.00114
14	0.00815			3.00	0.00157

**Table 3:** Relative errors between experimental and predicted values of TBARS, FFA, SSP, Ca^2+^-ATPase activity, and SH content at 253 K.

Parameters		Storage Time (Week)									
		1	2	3	5	7	9	11	13	15	17
TBARS	Predicted value	0.34	0.59	0.70	0.74	0.88	0.89	0.69	0.74	0.96	1.06
	Experimental value	0.34 ± 0.09	0.60 ± 0.11	0.70 ± 0.01	0.74 ± 0.02	0.88 ± 0.08	0.89 ± 0.04	0.69 ± 0.05	0.75 ± 0.08	0.96 ± 0.09	1.06 ± 0.11
	Relative errors (%)	−0.66	0.77	−0.52	−0.35	0.19	0.23	0.19	0.33	−0.00	0.19
FFA	Predicted value	5.23	7.42	9.48	11.18	12.02	13.82	14.14	14.79	15.54	16.82
	Experimental value	5.34 ± 0.96	7.06 ± 0.60	9.88 ± 0.33	10.90 ± 1.27	12.27 ± 1.43	13.61 ± 0.68	14.30 ± 4.35	14.69 ± 0.25	15.59 ± 1.29	16.81 ± 0.99
	Relative errors (%)	2.05	−5.16	4.05	−2.56	2.03	−1.56	1.11	−0.69	0.30	−0.06
SSP	Predicted value	58.79	58.88	57.82	58.82	58.90	57.56	55.80	46.28	41.49	43.17
	Experimental value	59.02 ± 5.57	58.10 ± 3.97	58.69 ± 4.48	58.22 ± 0.23	59.45 ± 1.92	57.08 ± 0.52	56.16 ± 1.03	46.05 ± 7.10	41.60 ± 10.39	43.14 ± 2.07
	Relative errors (%)	0.39	−1.34	1.47	−1.03	0.92	−0.83	0.64	−0.49	0.27	−0.07
Ca^2+^-ATP ase	Predicted value	0.24	0.20	0.19	0.17	0.13	0.10	0.05	0.04	0.03	0.06
	Experimental value	0.24 ± 0.01	0.20 ± 0.02	0.19 ± 0.02	0.17 ± 0.01	0.13 ± 0.00	0.09 ± 0.01	0.05 ± 0.01	0.04 ± 0.07	0.03 ± 0.01	0.05 ± 0.01
	Relative errors (%)	−0.38	−0.03	0.04	0.81	0.03	−4.92	3.35	−1.30	3.81	−9.18
SH	Predicted value	6.71	5.28	5.16	4.96	3.50	3.52	3.52	2.92	2.86	2.45
	Experimental value	6.71 ± 0.27	5.29 ± 0.18	5.15 ± 0.02	4.97 ± 0.78	3.49 ± 0.16	3.53 ± 0.34	3.52 ± 0.33	2.93 ± 0.76	2.85 ± 0.59	2.45 ± 0.09
	Relative errors (%)	−0.05	0.24	−0.26	0.18	−0.24	0.34	−0.21	0.27	−0.15	0.07

**Table 4:** MSE and R^2^ between experimental and predicted values for common carp fillets prediction of three frozen temperatures.

Parameters	Temperatures (K)	MSE	R^2^	Parameters	Temperatures (K)	MSE	R^2^
TBARS	261	0.000	1.000	Ca^2+^-ATPase activity	261	0.000	0.999
	253	0.000	1.000		253	0.000	0.999
	245	0.000	1.000		245	0.000	0.999
FFA	261	0.000	1.000	SH	261	0.000	1.000
	253	0.066	0.995		253	0.000	1.000
	245	0.000	1.000		245	0.000	1.000
SSP	261	0.001	1.000				
	253	0.308	0.994				
	245	0.001	1.000				

## Data Availability

Data are contained within the article.

## Supplementary Materials

The following supporting information can be downloaded at: https://www.mdpi.com/article/10.3390/foods12142741/s1, RBFNN: one hidden layer, twenty-eight hidden neurons.

## References

[1] Hao R., Pan J., Tilami S.K., Shah B.R., Mraz J. (2021). Post-mortem quality changes of common carp (*Cyprinus carpio*) during chilled storage from two culture systems. J. Sci. Food Agric..

[2] Setiadi E., Taufik I., Widyastuti Y.R., Ardi I., Saputra A. (2019). Different substrate of trickling filter on growth, survival rate, and water quality of common carp (*Cyprinus carpio*) cultivation by using an intensive recirculation system. IOP Conf. Ser. Earth Environ. Sci..

[3] FAO (2020). Understanding-Antimicrobial-Resistance-in-Aquaculture.

[4] FAO (2020). Fisheries Situation Report, January to December 2019.

[5] Di Renzo L., Gualtieri P., Pivari F., Soldati L., Attina A., Cinelli G., Leggeri C., Caparello G., Barrea L., Scerbo F. (2020). Eating habits and lifestyle changes during COVID-19 lockdown: An Italian survey. J. Transl. Med..

[6] Walayat N., Rincón M.Á., Niaz S., Nawaz A., Niaz N., Zahid Farooq M., Ahmad I., Wang P., Zhang Z. (2021). Egg white proteins and β-cyclodextrin: Effective cryoprotectant mixture against oxidative changes in the myofibrillar proteins of *Culter alburnus*. Int. J. Food Sci. Technol..

[7] Trigo M., Rodríguez A., Dovale G., Pastén A., Vega-Gálvez A., Aubourg S.P. (2018). The effect of glazing based on saponin-free quinoa (*Chenopodium quinoa*) extract on the lipid quality of frozen fatty fish. LWT.

[8] Carrera M., Fidalgo L.G., Vazquez M., Saraiva J.A., Aubourg S.P. (2020). Comparative effect of a previous 150-MPa treatment on the quality loss of frozen hake stored at different temperatures. J. Sci. Food Agr..

[9] Jiang L., Wu S. (2018). Pullulan suppresses the denaturation of myofibrillar protein of grass carp (*Ctenopharyngodon idella*) during frozen storage. Int. J. Biol. Macromol..

[10] Tatiyaborworntham N., Oz F., Richards M.P., Wu H. (2022). Paradoxical effects of lipolysis on the lipid oxidation in meat and meat products. Food Chem. X.

[11] Masniyom P. (2011). Deterioration and shelf-life extension of fish and fishery products by modified atmosphere packaging. Songklanakarin J. Sci. Technol..

[12] Aubourg S.P. (2016). Review: Loss of Quality during the Manufacture of Canned Fish Products. Food Sci. Technol. Int..

[13] Hematyar N., Masilko J., Mraz J., Sampels S. (2018). Nutritional quality, oxidation, and sensory parameters in fillets of common carp (*Cyprinus carpio* L.) influenced by frozen storage (−20 °C). J. Food Process. Preserv..

[14] Van Hecke T., Goethals S., Vossen E., De Smet S. (2019). Long-Chain n-3 PUFA Content and n-6/n-3 PUFA Ratio in Mammal, Poultry, and Fish Muscles Largely Explain Differential Protein and Lipid Oxidation Profiles Following In Vitro Gastrointestinal Digestion. Mol. Nutr. Food Res..

[15] Hematyar N., Rustad T., Sampels S., Kastrup Dalsgaard T. (2019). Relationship between lipid and protein oxidation in fish. Aquac. Res..

[16] Zhang M., Li F., Diao X., Kong B., Xia X. (2017). Moisture migration, microstructure damage and protein structure changes in porcine longissimus muscle as influenced by multiple freeze-thaw cycles. Meat Sci..

[17] Wang Y., Miyazaki R., Saitou S., Hirasaka K., Takeshita S., Tachibana K., Taniyama S. (2018). The effect of ice crystals formations on the flesh quality of frozen horse mackerel (*Trachurus japonicus*). J. Texture Stud..

[18] Tian J., Walayat N., Ding Y., Liu J. (2022). The role of trifunctional cryoprotectants in the frozen storage of aquatic foods: Recent developments and future recommendations. Compr. Rev. Food Sci. Food Saf..

[19] Ke P.J., Cervantes E., Robles-Martinez C. (1984). Determination of Thiobarbituric Acid Reactive Substances (TBARS) in Fish Tissue by an Improved Distillation-Spectrophotometric Method. J. Sci. Food Agric..

[20] Sowa-Kucma M., Styczen K., Siwek M., Misztak P., Nowak R.J., Dudek D., Rybakowski J.K., Nowak G., Maes M. (2018). Lipid Peroxidation and Immune Biomarkers Are Associated with Major Depression and Its Phenotypes, Including Treatment-Resistant Depression and Melancholia. Neurotox. Res..

[21] Zeng N., Huang R., Li N., Jiang H., Li R., Wang F., Chen W., Xia M., Wang Q. (2018). MiR-451a attenuates free fatty acids-mediated hepatocyte steatosis by targeting the thyroid hormone responsive spot 14 gene. Mol. Cell Endocrinol..

[22] Pavillard L.E., Marin-Aguilar F., Bullon P., Cordero M.D. (2018). Cardiovascular diseases, NLRP3 inflammasome, and western dietary patterns. Pharmacol. Res..

[23] Lee K.R., Midgette Y., Shah R. (2019). Fish Oil Derived Omega 3 Fatty Acids Suppress Adipose NLRP3 Inflammasome Signaling in Human Obesity. J. Endocr. Soc..

[24] Andevari G.T., Rezaei M. (2011). Effect of gelatin coating incorporated with cinnamon oil on the quality of fresh rainbow trout in cold storage. Int. J. Food Sci. Technol..

[25] Yin C., Wang J., Qian J., Xiong K., Zhang M. (2022). Quality changes of rainbow trout stored under different packaging conditions and mathematical modeling for predicting the shelf life. Food Packag. Shelf Life.

[26] Wang H., Zheng Y., Shi W., Wang X. (2022). Comparison of Arrhenius model and artificial neuronal network for predicting quality changes of frozen tilapia (*Oreochromis niloticus*). Food Chem..

[27] Chen S., Tao F., Pan C., Hu X., Ma H., Li C., Zhao Y., Wang Y. (2020). Modeling quality changes in Pacific white shrimp (*Litopenaeus vannamei*) during storage: Comparison of the Arrhenius model and Random Forest model. J. Food Process. Preserv..

[28] Xu Z., Liu X., Wang H., Hong H., Luo Y. (2017). Comparison between the Arrhenius model and the radial basis function neural network (RBFNN) model for predicting quality changes of frozen shrimp (*Solenocera melantho*). Int. J. Food Prop..

[29] Li Q., Xiong Q., Ji S., Yu Y., Wu C., Yi H. (2021). A method for mixed data classification base on RBF-ELM network. Neurocomputing.

[30] Yu J., Zhan J., Huang W. (2017). Identification of Wine According to Grape Variety Using Near-Infrared Spectroscopy Based on Radial Basis Function Neural Networks and Least-Squares Support Vector Machines. Food Anal. Methods.

[31] Yang X., Liu Y., Chen J., Lv Y., Luo Y. (2018). Quality Attributes and Shelf Life Modeling of Pacific White Shrimp (*Litopenaeus vannamei*) Stored at Different Temperatures. J. Aquat. Food Prod. Technol..

[32] Geng Z., Liu F., Shang D., Han Y., Shang Y., Chu C. (2021). Early warning and control of food safety risk using an improved AHC-RBF neural network integrating AHP-EW. J. Food Eng..

[33] Noori S.M.A., Khanzadi S., Fazlara A., Najafzadehvarzi H., Azizzadeh M. (2018). Effect of lactic acid and ajwain (*Carum copticum*) on the biogenic amines and quality of refrigerated common carp (*Cyprinus carpio*). Lwt.

[34] Kong C., Wang H., Li D., Zhang Y., Pan J., Zhu B., Luo Y. (2016). Quality changes and predictive models of radial basis function neural networks for brined common carp (*Cyprinus carpio*) fillets during frozen storage. Food Chem..

[35] Shi J., Lei Y., Shen H., Hong H., Yu X., Zhu B., Luo Y. (2019). Effect of glazing and rosemary (*Rosmarinus officinalis*) extract on preservation of mud shrimp (*Solenocera melantho*) during frozen storage. Food Chem..

[36] Liu Y., Zhang L., Gao S., Bao Y., Tan Y., Luo Y., Li X., Hong H. (2022). Effect of protein oxidation in meat and exudates on the water holding capacity in bighead carp (*Hypophthalmichthys nobilis*) subjected to frozen storage. Food Chem..

[37] Yu Q., Liu J., Liu Y., Zheng Y., Pi R., Mubango E., Tan Y., Luo Y., Hong H. (2022). Inhibitive effect of cryoprotectants on the oxidative and structural changes in myofibrillar proteins of unwashed mince from silver carp during frozen storage. Food Res. Int..

[38] Lin J., Hong H., Zhang L., Zhang C., Luo Y. (2019). Antioxidant and cryoprotective effects of hydrolysate from gill protein of bighead carp (*Hypophthalmichthys nobilis*) in preventing denaturation of frozen surimi. Food Chem..

[39] Zheng Y., Zhou F., Zhang L., Wang H., Wang X.-c. (2021). Effect of different extent of protein oxidation on the frozen storage stability of muscle protein in obscure pufferfish (*Takifugu obscurus*). Lwt.

[40] Han H.G., Chen Q.L., Qiao J.F. (2011). An efficient self-organizing RBF neural network for water quality prediction. Neural Netw..

[41] Zhang H., Zhang Y., Javed M., Cheng M., Xiong S., Liu Y. (2021). Gelatin hydrolysates from sliver carp (*Hypophthalmichthys molitrix*) improve the antioxidant and cryoprotective properties of unwashed frozen fish mince. Int. J. Food Sci. Technol..

[42] Klein R.D., Rosa C.E., Colares E.P., Robaldo R.B., Martinez P.E., Bianchini A. (2017). Antioxidant defense system and oxidative status in Antarctic fishes: The sluggish rockcod *Notothenia coriiceps* versus the active marbled notothen Notothenia rossii. J. Therm. Biol..

[43] Yu Y.J., Yang S.P., Lin T., Qian Y.F., Xie J., Hu C. (2020). Effect of Cold Chain Logistic Interruptions on Lipid Oxidation and Volatile Organic Compounds of Salmon (*Salmo salar*) and Their Correlations With Water Dynamics. Front. Nutr..

[44] Aguiar Saldanha Pinheiro A.C., Tappi S., Patrignani F., Lanciotti R., Romani S., Rocculi P. (2020). Effects of novel modified atmosphere packaging on lipid quality and stability of sardine (*Sardina pilchardus*) fillets. Int. J. Food Sci. Technol..

[45] Zhu Z., Zhou Q., Sun D.-W. (2019). Measuring and controlling ice crystallization in frozen foods: A review of recent developments. Trends Food Sci. Technol..

[46] Jia H., Roy K., Pan J., Mraz J. (2022). Icy affairs: Understanding recent advancements in the freezing and frozen storage of fish. Compr. Rev. Food Sci. Food Saf..

[47] Li T., Kuang S., Xiao T., Hu L., Nie P., Ramaswamy H.S., Yu Y. (2022). The Effect of Pressure-Shift Freezing versus Air Freezing and Liquid Immersion on the Quality of Frozen Fish during Storage. Foods.

[48] Tanaka R., Nakazawa N., Fukushima H., Watanabe M., Maekawa K., Okano T., Hiraishi K., Okazaki E. (2021). Effects of Initial Freshness Level, Frozen Storage Temperature, and Storage Period on Lipid Deterioration and K-value in Meat Blocks from Chub Mackerel *Scomber japonicus*. J. Aquat. Food Prod. Technol..

[49] Bejaoui S., Ghribi F., Chetoui I., Aouini F., Bouaziz M., Houas-Gharsallah I., Soudani N., El Cafsi M. (2021). Effect of storage temperature and time on the fatty acids and nutritional quality of the commercial mussel (*Mytilus galloprovincialis*). J. Food Sci. Technol..

[50] Gomez-Estaca J., Gomez-Guillen M.C., Marin-Penalver D., Montero M.P. (2020). Functional aptitude of hake minces with added TMAO-demethylase inhibitors during frozen storage. Food Chem..

[51] Shi L., Yang T., Xiong G., Li X., Wang X., Ding A., Qiao Y., Wu W., Liao L., Wang L. (2018). Influence of frozen storage temperature on the microstructures and physicochemical properties of pre-frozen perch (*Micropterus salmoides*). LWT.

[52] Gullian-Klanian M., Terrats-Preciat M., Pech-Jiménez E.C., Cutz De Ocampo J. (2017). Effect of Frozen Storage on Protein Denaturation and Fatty Acids Profile of the Red Octopus (*Octopus maya*). J. Food Process. Preserv..

[53] Odoli C.O., Oduor-Odote P., Arason S. (2019). The influence of lipid content and pretreatment methods on protein conformation in fish (capelin, *Mallotus villosus*) during smoking and drying. Food Sci. Nutr..

[54] Sriket P., La-ongnual T. (2018). Quality Changes and Discoloration of Basa (*Pangasius bocourti*) Fillet during Frozen Storage. J. Chem..

[55] Wu H., Wang Z., Luo Y., Hong H., Shen H. (2014). Quality Changes and Establishment of Predictive Models for Bighead Carp (*Aristichthys nobilis*) Fillets During Frozen Storage. Food Bioprocess. Technol..

[56] Konno K. (2017). Myosin Denaturation Study for the Quality Evaluation of Fish Muscle-based Products. Food Sci. Technol. Res..

[57] Welle M., Pedersen J.T., Ravnsborg T., Hayashi M., Maass S., Becher D., Jensen O.N., Stohr C., Palmgren M. (2021). A conserved, buried cysteine near the P-site is accessible to cysteine modifications and increases ROS stability in the P-type plasma membrane H+-ATPase. Biochem. J..

[58] Zhao X., Zhou Y., Zhao L., Chen L., He Y., Yang H. (2019). Vacuum impregnation of fish gelatin combined with grape seed extract inhibits protein oxidation and degradation of chilled tilapia fillets. Food Chem..

[59] Cai L., Nian L., Cao A., Zhang Y., Li X. (2019). Effect of Carboxymethyl Chitosan Magnetic Nanoparticles Plus Herring Antifreeze Protein on Conformation and Oxidation of Myofibrillar Protein From Red Sea Bream (*Pagrosomus major*) After Freeze-Thaw Treatment. Food Bioprocess. Technol..

[60] Gao W., Huang Y., Zeng X.A., Brennan M.A. (2019). Effect of soluble soybean polysaccharides on freeze-denaturation and structure of myofibrillar protein of bighead carp surimi with liquid nitrogen freezing. Int. J. Biol. Macromol..

[61] Qian P., Zhang Y., Shen Q., Ren L., Jin R., Xue J., Yao H., Dai Z. (2018). Effect of cryogenic immersion freezing on quality changes of vacuum-packed bighead carp (*Aristichthys nobilis*) during frozen storage. J. Food Process. Preserv..

[62] Lu H., Liang Y., Zhang L., Shi J. (2021). Modeling relationship between protein oxidation and denaturation and texture, moisture loss of bighead carp (*Aristichthys Nobilis*) during frozen storage. J. Food Sci..

[63] Gao S., Liu Y., Fu Z., Zhang H., Zhang L., Li B., Tan Y., Hong H., Luo Y. (2023). Uncovering quality changes of salted bighead carp fillets during frozen storage: The potential role of time-dependent protein denaturation and oxidation. Food Chem..

[64] Kaymak-Ertekin F., Gedik A. (2005). Kinetic modelling of quality deterioration in onions during drying and storage. J. Food Eng..

