# Supplementary material for: Changes in Lipids and Proteins of Common Carp (Cyprinus carpio) Fillets under Frozen Storage and Establishment of a Radial Basis Function Neural Network (RBFNN)
Source: Foods. 2023 Jul 19;12(14):2741. doi: 10.3390/foods12142741 (PMC10379316; doi:10.3390/foods12142741)
Supplement: Supplementary file 1 [file foods-12-02741-s001.zip › foods-2366792-supplementary.pdf]

Supplementary Material

RBFNN: one hidden layer, twenty-eight hidden neurons.

Input:

$$X_1 = \frac{\text{Temperature} - 253}{8} \quad (245\ \text{K} < \text{Temperature} < 261\ \text{K})$$

$$X_2 = \frac{\text{Time} - 9}{8} \quad (\text{Time} > 0\ \text{week})$$

Radial basis layer:

$$X_{p(p=3,4\ldots30)} = exp\left[-\left[\sqrt{\sum[\mathrm{w_{ji}} - \mathrm{x_i}]^2} \times \mathrm{w_{j0}}\right]^2\right] = \exp\left[-0.8326^2 \times \left[\frac{\| \mathrm{w_{ji}} - \mathrm{x_i} \|}{1.67}\right]^2\right]$$

$$\mathrm{w_{ji}} = \begin{bmatrix} 1.00 & -1.00 & 0.00 & 0.00 & -1.00 & 1.00 & -1.00 & 1.00 & 0.00 & 0.00 & -1.00 \\ 0.75 & -0.50 & 0.50 & -1.00 & 0.75 & -1.00 & -1.00 & 0.50 & 0.25 & 0.75 & 1.00 \end{matrix}$$

$$\begin{matrix} 1.00 & 1.00 & 1.00 & -1.00 & -1.00 & 0.00 & 0.00 & 0.00 & 0.00 & 1.00 & 1.00 \\ 1.00 & -0.88 & -0.75 & -0.88 & -0.75 & -0.88 & -0.75 & -0.50 & 0.00 & -0.50 & -0.25 \end{matrix}$$

$$\begin{matrix} -1.00 & 1.00 & 1.00 & -1.00 & -1.00 & -1.00 \\ -0.25 & 0.25 & 0.00 & 0.00 & 0.50 & 0.25 \end{bmatrix}^T$$

Output layer:

$$X_{q(q=31,32,33,34,35)} = \sum w_{kj} X_j + w_{k0}$$

$$w_{kj} = \begin{bmatrix} -229.73 & -346.34 & 5.46 & -29.70 & -65.15 & -835.00 & 249.71 \\ -192.19 & 66.57 & 3.98 & 1.43 & -15.26 & -258.34 & -23.95 \\ 92.57 & 538.89 & -32.76 & -34.75 & -68.66 & 598.55 & -239.81 \\ 50.91 & 2716.07 & 40.46 & 79.84 & -421.46 & 182.16 & -1158.64 \\ -114.83 & -788.66 & 13.33 & 193.10 & 108.73 & -78.23 & 450.16 \end{matrix}$$

$$\begin{matrix} 489.98 & -9.82 & -1.11 & 23.08 & 62.81 & 2611.53 & -2697.111 & -751.38 \\ 380.40 & -5.80 & -1.72 & 3.32 & 56.38 & 893.17 & -1023.95 & 76.74 \\ -230.69 & 44.03 & 14.80 & 23.57 & -18.33 & -1818.30 & 1837.06 & 800.90 \\ -103.36 & -55.55 & -20.15 & 104.81 & -20.49 & -602.06 & 633.69 & 3777.78 \\ 244.78 & -29.03 & -2.09 & -28.27 & 31.05 & 387.04 & -538.83 & -1351.52 \end{matrix}$$

$$\begin{matrix} 724.02 & 57.22 & -31.54 & 0.58 & 7.08 & 1661.38 & -1371.25 \\ -86.57 & -12.02 & 14.53 & -6.58 & 4.30 & 772.63 & -764.05 \\ -854.74 & 117.86 & -112.32 & 43.74 & -29.76 & -1084.15 & 855.22 \\ -4072.98 & -231.35 & 201.03 & -67.81 & 39.30 & -392.69 & 311.98 \\ 1349.20 & -474.94 & 368.45 & -94.73 & 34.13 & 34.13 & -520.88 \end{matrix}$$

$$\begin{matrix} 168.90 & -793.75 & 1095.95 & -22.16 & 93.42 & -70.09 \\ -69.37 & -568.46 & 705.39 & 65.33 & 32.80 & -52.47 \\ -433.38 & 425.57 & -639.61 & 344.15 & 142.75 & -231.01 \\ -2399.74 & 164.16 & -240.64 & 2008.53 & 916.02 & -1491.15 \\ 635.76 & -382.74 & 485.69 & -507.37 & -231.32 & 372.18 \end{bmatrix}$$

$$w_{k0} = \begin{bmatrix} -0.0789 \\ 0.1692 \\ -4.2737 \\ 6.0797 \\ -1.8598 \end{bmatrix}$$

$$\triangle TBARS = 0.61X31 + 0.67$$
$$\triangle FFA = 17.11X32 + 18.02$$
$$\triangle SSP = 19.92X33 - 21.47$$
$$\triangle Ca^{2+} - ATPase = 0.12X34 - 0.13$$
$$\triangle SH = 2.20X35 - 2.47$$
